# Impact of Power Converter Current Ripple on the Degradation of PEM Electrolyzer Performances

**DOI:** 10.3390/membranes12020109

**Published:** 2022-01-19

**Authors:** François Parache, Henri Schneider, Christophe Turpin, Nicolas Richet, Olivier Debellemanière, Éric Bru, Anh Thao Thieu, Caroline Bertail, Christine Marot

**Affiliations:** 1 Laboratoire Plasma et Conversion d’énergie Université de Toulouse (LAPLACE), Centre National de la Recherche Scientifique (CNRS), Institut National Polytechnique de Toulouse(INPT), Université Paul Sabatier (UPS), 31077 Toulouse, France; schneide@laplace.univ-tlse.fr (H.S.); turpin@laplace.univ-tlse.fr (C.T.); bru@laplace.univ-tlse.fr (É.B.); 2 Air Liquide, Research & Development, Paris Innovation Campus, 1 Chemin de la Porte des Loges, 78354 Jouy en Joses, France; nicolas.richet@airliquide.com (N.R.); olivier.debellemaniere@airliquide.com (O.D.); anhthao.thieu@airliquide.com (A.T.T.); caroline.bertail@airliquide.com (C.B.); christine.marot@airliquide.com (C.M.)

**Keywords:** PEM electrolyzer, ripple current, degradation

## Abstract

In this study, an endurance test of 3000 h was conducted on four equivalent proton exchange membrane (PEM) electrolyzers to identify and quantify the impact of an electric ripple current on their durability. Three different typical power converter waveforms and frequencies were explored. Signals were added to the same direct current carrier and also tested for reference. Performance comparison based on polarization curves and electrochemical impedance spectroscopy (EIS) analysis revealed that the ripple current favors degradation. Triangular waveform and a frequency of 10 kHz were identified as the most degrading conditions, leading to a sharp increase in high-frequency resistance (HFR) and the emergence of mass transport limitations due to the enhanced degradation of titanium mesh. Moreover, reversible losses were observed and further explorations are needed to decorrelate them from our observations.

## 1. Introduction

With the ambition to reduce fossil energy consumption and to mitigate climate change, the use of renewable energy sources (RES) is rapidly expanding, promoted by multiple policies [1]. However, these sources are constrained by their availability and intermittence, and cannot be used to provide steady energy whenever it is needed. Energy storage is, therefore, necessary to achieve electrical network stability. Hydrogen, as a very efficient energy vector, is a promising option for solving these issues [2,3]. The use of electrolyzer flexible operating capability has also been considered for power quality improvement and grid frequency regulation [4,5]. With a view to reducing greenhouse gas emissions, hydrogen strategies only make sense in the case of green hydrogen production. This has resulted in a growing trend for the use of water electrolysis associated with RES [6]. There are already many examples of this pairing, and this fast development will definitely shake the global energy market in the future [7,8,9].

Proton exchange membrane water electrolyzers (PEMWEs) are usually used for these applications. Their capacity to operate at high current densities [10] and the purity of the gas produced [11,12] enable this technology to be adapted for large-scale production. They are also renowned for their dynamic operation [13,14], which constitutes an advantage for coupling to intermittent RES. However, running under such variations may have an impact on the component efficiency and durability. Buitendach et al. demonstrated that power consumption rises when the ripple factor is increased for any waveform [15]. On the contrary, Frensch et al. observed performance enhancement during 500 h of fast cycling operation due to a decrease in ohmic resistance but also showed potential degradation [16]. Even if dynamic operation is identified as a key factor for an accelerated stress test (AST), some tests show no degradation, especially with square current waveforms [5,17]. Rakousky et al. presented degradant behavior for short interruptions. They also showed that periodically reducing or stopping the current may instead improve durability [13]. Thus, it is difficult to conclude the impact of dynamic operation and waveforms on electrolyzers.

Other current fluctuations are found in electrolysis systems. Indeed, power converters are usually used in various autonomous or grid-connected applications [14,18]. They can be advantageous for some systems [19] and provide adaptability to variable conditions [20,21]. DC/DC converters can be associated with photovoltaic sources, and AC/DC rectifiers can be used for grid coupling. The switching operation of converters then induces output current ripples [22]. Usually, studies try to achieve current ripple reduction. Various topologies have already been tested for this purpose [23,24]. Ripple currents, topologies, and control strategies have a direct impact on the efficiency and power quality of electrolysis systems [15,23,25,26,27]. However, these studies have not taken into account possible long-term degradation.

Concerning the influence of ripple current on component lifespan, numerous investigations have been carried out on fuel cells. Many of these have alerted of a potential lifespan reduction [28,29,30]. The effect of undulations can be closely linked to their frequency. Low-frequency ripple currents (<100 Hz) are likely to reduce Pt/C catalyst durability [31,32], whereas at higher frequencies (up to 1 kHz) the impact on lifespan is more tenuous [31,33].

Even if similar consequences can be presumed on PEM electrolyzers [34], to the best of the authors’ knowledge, no equivalent studies have been performed so far. Yet, manufacturers usually put major constraints on input current quality, which affects the size and the price of the power supply system. Indeed, power supply represents more than a quarter of the cost of a PEM electrolysis system. Thus, it is identified as the largest area where cost reduction can be achieved [1]. More specifically, power electronics are estimated to contribute up to 15% of the total cost [35]. The present work aims to study the impact of current ripple on PEM electrolyzers’ durability in order to clarify whether the constraints on their sizing are necessary or can be alleviated.

## 2. Materials and Methods

### 2.1. Materials and Setup

The PEM electrolyzer cells (PEMECs) investigated in this study were dual-cell electrolyzers of 16 cm^2^ from H-Tec Education (College Station, TX, USA). IrOx blend was used at the anode, while the cathode was loaded with 60% Pt/C. A titanium mesh was used as a flow-field and current collector for both electrodes.

**Figure 1:**
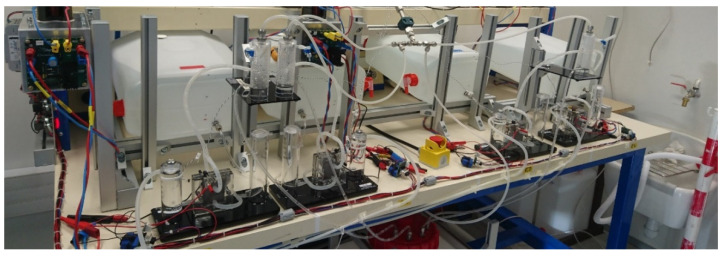
Test bench with four H-Tec PEM “Electrolyzer H_2_/O_2_ 65”.

**Figure 2:**
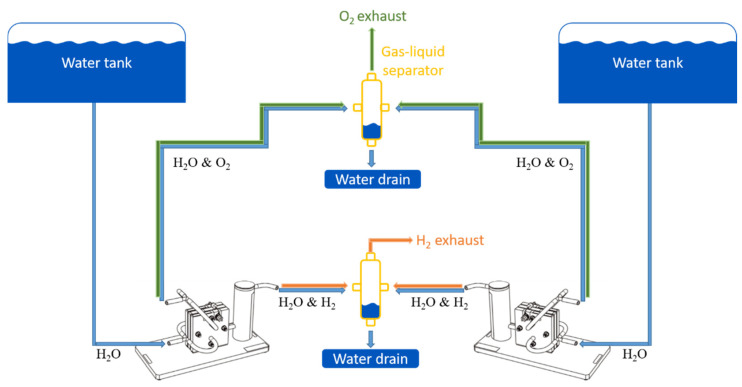
Schematic overview of fluid connections.

In order to allow 24/7 operation, a 20 L deionized water tank was set up to supply the process with a gravity flow at the anode input of each electrolyzer. Waste water dragged by the gases produced was not recycled to avoid any contaminant accumulation. Thus, necessary refueling was scheduled twice a week, allowing maintenance of a high renewal rate and the control of water conductivity (under 0.86 μS·cm^−2^) (Figure 1 and Figure 2).

**Figure 3:**
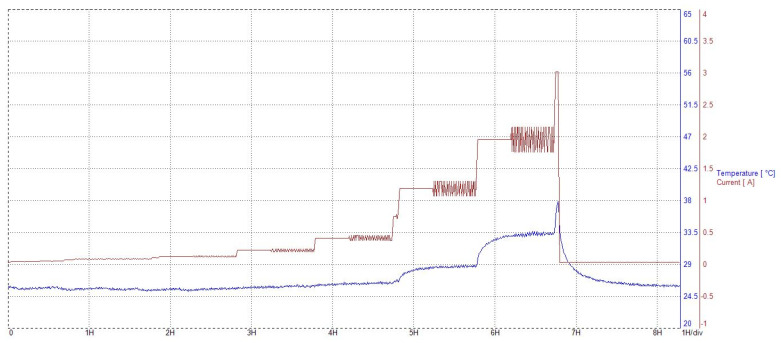
Example of temperature variations during characterization phases.

Parallel to the imposed current profiles, stack voltage and temperature were constantly recorded for each electrolyzer as well as the room temperature. All components were operated at ambient temperature (regulated at 25 °C) and atmospheric pressure. During operation, the input anode pressure varied a little due to the water consumption, resulting in the depletion of the tank (maximum variation of 10 mbar). Since the components’ temperature was not regulated, it could increase with the current density during the characterization phases (Figure 3).

Safety limitations were set for high temperatures to prevent thermal runaway, and for low currents to shut down all running electrolyzers if one of them were to abruptly stop. A limit voltage of 2 V was also chosen for the cells, resulting in a relatively low and protective operating point in both voltage and current. This choice ensured that the observed degradations originated from ripple current rather than other operating conditions.

**Figure 4:**
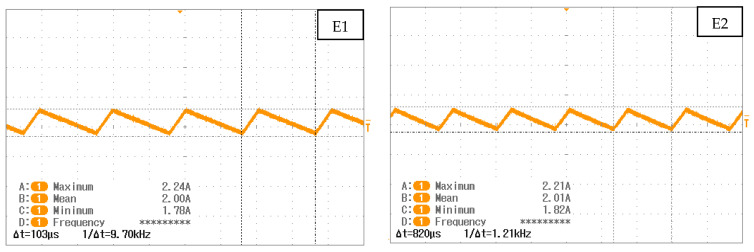

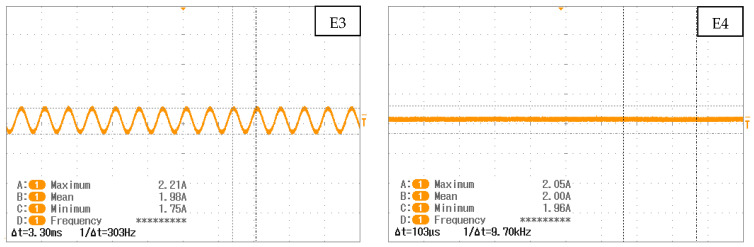
Observation of power supply waveform for each electrolyzer. The frequency is not displayed directly but is shown as the inverse of the period.

The durability experiment presented in this paper consisted of running PEM electrolyzers for 3000 h with ripple currents characteristic of converters. Two triangular ripple currents of 10 kHz and 1 kHz were generated by step-down converters and were imposed on electrolyzers E1 and E2, respectively. The third component, E3, was stressed with a 300 Hz sinusoidal ripple current, provided by a dynamic power supply. In each case, ripple currents were around 2 A (0.125 A/cm^2^) with an amplitude of +/−10%. To analyze and compare the results, a reference test at 2 A constant current was also carried out on E4 (Figure 4).

### 2.2. Electrochemical Characterization

Characterization phases were set every 500 h, after a short stop period of one hour, to limit the observation of reversible phenomena and highlight irreversible losses. Indeed, reversible losses were observed during operation and will be discussed later in this article.

The PEMECs were characterized with iV-curve and electrochemical impedance spectroscopy (EIS) for each current step of the polarization curve. Depending on the current level, two types of EIS were created. “Complete” EIS was used for six specific steps and explored a large frequency range from 10 kHz to 10 mHz. For every other step, EIS was restricted from 10 kHz to 1 Hz. For both of these, the alternating current was defined as 10% of the current step. Results were presented in a Nyquist plot, where the ohmic resistance was identified on the impedance spectrum at high-frequency intersection with the real axis. It included the electronic contact resistance as well as the membrane’s protonic resistance and is termed HFR in this manuscript.

## 3. Results and Discussion

### 3.1. Initial Characterizations

First of all, an analysis of initial characterizations was performed. It appeared quite important to study the original state differences between the four electrolyzers in order to compare their degradation after operation.

#### 3.1.1. Initial Polarization Curves

**Figure 5:**
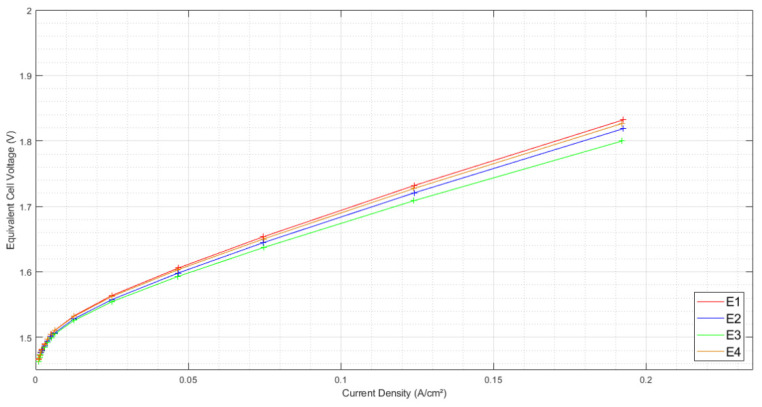
Initial polarization curve for each electrolyzer, from 0 to 0.19 A/cm^2^, at ambient pressure of 1 bar, with an ambient temperature regulated at 25 °C.

Figure 5 shows the initial polarization curves together. The equivalent cell voltage is presented as a function of the current density.

A small disparity was observed between the performances of the four stacks in their initial state. Those slight differences seemed partly related to slope inequalities. This can probably be attributed to ohmic resistance disparities.

#### 3.1.2. Initial EIS

**Figure 6:**
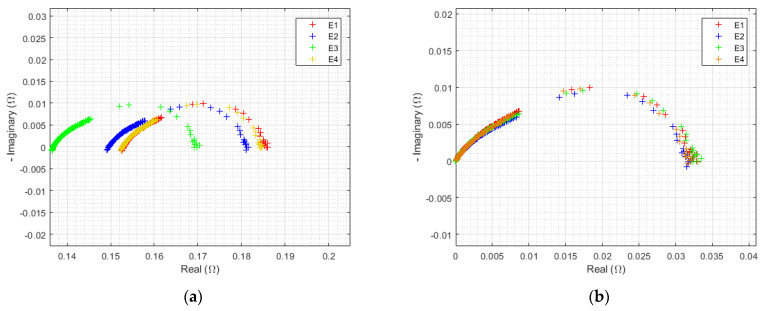
Initial EIS at 0.125 A/cm^2^ from 10 kHz to 10 mHz for each electrolyzer, at ambient pressure of 1 bar, with an ambient temperature regulated at 25 °C: (**a**) not altered, (**b**) with HFR subtracted.

As for every electrochemical component, activation losses are observable by EIS. These are commonly expressed in models as a logarithmic function of current [36]. Thus, they are minimized in proportion at the uppermost current density. This condition, therefore, was chosen to better observe ohmic resistance disparities (Figure 6a). The HFR differences, previously mentioned, could then be recognized. If the HFR value is subtracted from the real part of the impedance for each point of the spectrum, the visual comparison of the resulting semicircles is made easier. Uniform results were found (Figure 6b).

**Figure 7:**
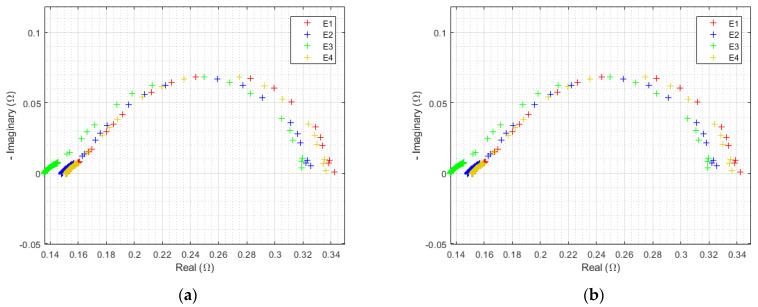
Initial EIS at 0.0125 A/cm^2^ from 10 kHz to 10 mHz for each electrolyzer, at ambient pressure of 1 bar, with an ambient temperature regulated at 25 °C: (**a**) not altered, (**b**) with HFR subtracted.

On the contrary, charge transfer phenomena were predominant at lower current densities. Figure 7 presents initial EIS at 0.0125 A/cm^2^. In addition, HFR differences were observed (Figure 7a) with a particular signature for the E3 electrolyzer, whereas charge transfer semicircles were quite homogeneous.

Thus, ohmic resistance disparities were considered responsible for the initial performance difference between the components tested. This analysis explains the better initial performance of electrolyzer E3. This is inherent to the electrolyzers’ assembly and should not bias our degradation analysis.

### 3.2. Characterization after 3000 h of Operation

#### 3.2.1. Polarization Curves at 3000 h of Aging

**Figure 8:**
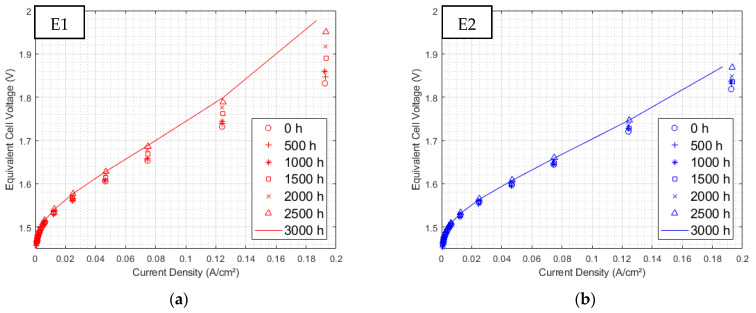

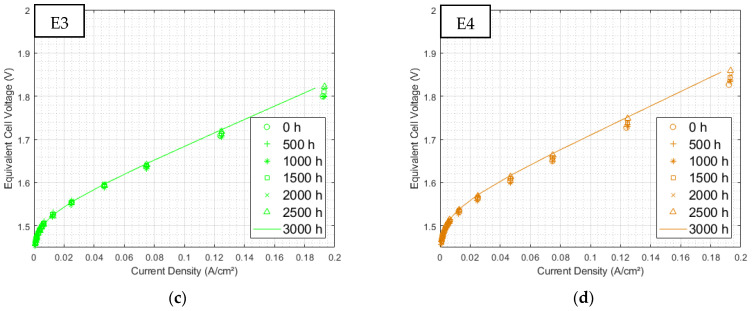
Evolution of polarization curves over time for electrolyzer: (**a**) E1, (**b**) E2, (**c**) E3, and (**d**) E4.

After 3000 h of operation, all electrolyzers displayed a clear overvoltage (Figure 8).

Table 1 presents the stack overvoltage evolution rates for the highest polarization current density (0.19 A/cm^2^). While electrolyzer E3, supplied with 300 Hz sinusoidal current ripple, does not seem particularly degraded with regard to the evolution of the reference component, both electrolyzers E1 and E2, withstanding triangular current ripple, deteriorated and presented overvoltage with increasing rates almost 5 and 1.8 times, respectively, higher than that of the reference.

In addition, after 3000 h the E1 polarization curve showed a slope change at 0.125 A/cm^2^. This probably results from mass transport complications, which are more likely to occur at high current densities. This hypothesis will be further explicated in the analysis of EIS.

#### 3.2.2. EIS at 3000 h

**Figure 9:**
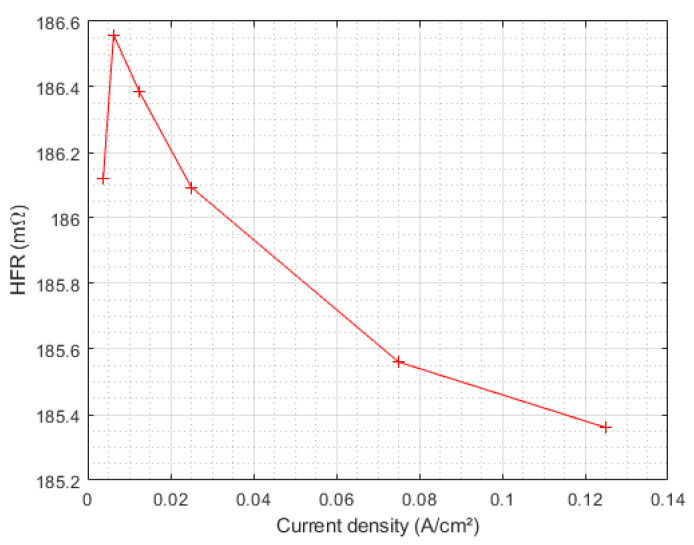
HFR evolution with the current during the characterization of E1 after 3000 h of operation.

As described in Section 2.2, HFR is identified on the impedance spectrum as the high-frequency intersection with the real axis. This measure fluctuates very little with the current density (Figure 9), even for the most degraded electrolyzer after 3000 h of operation. It is, thus, considered independent of the current density and will be determined only for 0.125 A/cm^2^.

**Figure 10:**
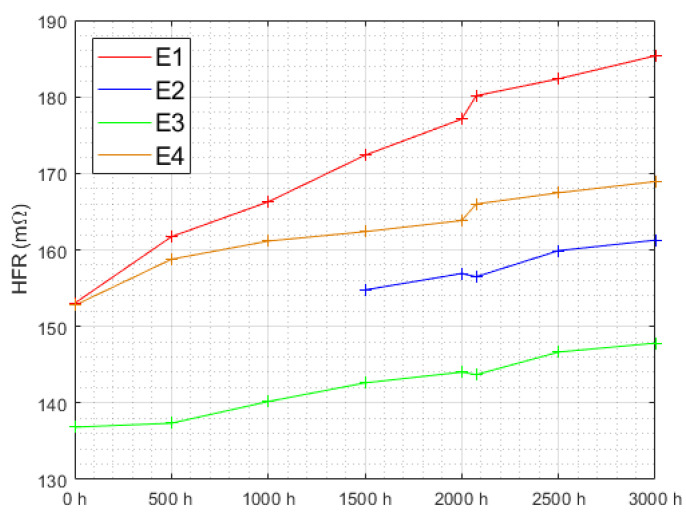
HFR evolution over time for all electrolyzers. For electrolyzer E2, the first three points were not coherent and are not presented here.

Figure 10 displays the evolution of HFR during the entire experiment. Once again, E1, withstanding triangular current ripple at 10 kHz, stands out. Its HFR increased twice as much as any other in this study after a linear fitting (Table 2).

Such a raise in the HFR value can have numerous origins. A porous transport layer (PTL) surface roughness, and high porosity [37], as well as catalyst–ionomer segregation and non-uniformities, are likely to increase ohmic losses [38]. More specifically, reactions at the anode with hydrogen crossing over the membrane can lead to the dissolution and re-precipitation of iridium within the membrane. However, this phenomenon is stimulated during voltage cycles with a large amplitude [39]. Operating conditions imposed here do not lead to such phenomena. Additionally, membrane dehydration results in a similar effect [40]. However, no water supply problem was observed in our case and the HFR value was stable with respect to current, while such dehydration complications would have increased with the current density.

Water quality also has an impact on ohmic resistance because of cationic impurities that can contaminate the membrane. This poisoning reduces the membrane conductivity and increases ohmic resistance but can also raise charge transfer resistance [41]. The water renewal system and quality control that have been put in place should be sufficient to avoid ionic contamination. However, cations can also come from cell component degradation, such as flow-fields or PTL corrosion, which also introduce mass transport complications.

The leading hypothesis is titanium passivation in the presence of oxygen at the anode side. This insulating layer on the titanium surface increases the contact resistance [42,43,44]; this measurement increase should be further investigated to test this phenomenon [39,45,46], which was not realized in this study.

**Figure 11:**
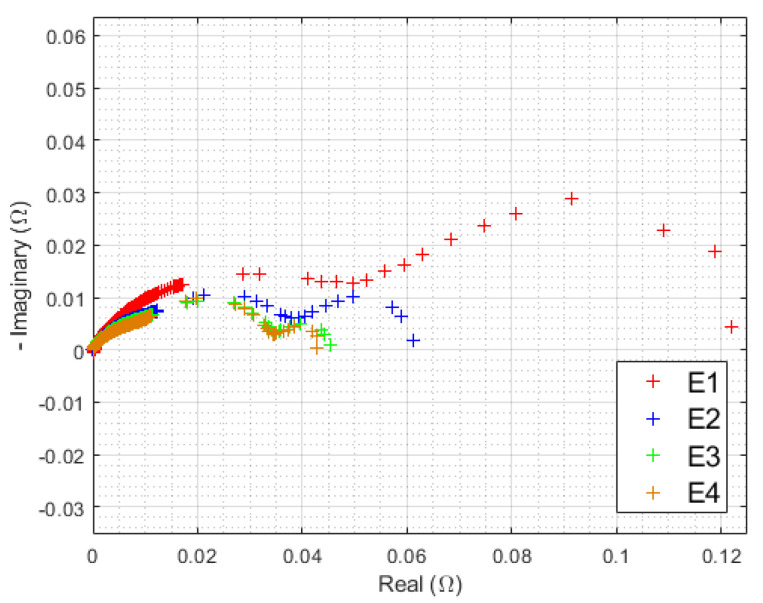
EIS at 0.125 A/cm^2^ from 10 kHz to 10 mHz of each electrolyzer after 3000 h of operation, at ambient pressure of 1 bar, with an ambient temperature regulated at 25 °C, HFR value is subtracted from the real part of each impedance data point.

HFR evolution has already been studied and will be systematically subtracted from each spectrum. Although they are almost identical at the initial state, EIS signature at maximal current density diverges significantly after 3000 h of operation. This fact is highlighted in Figure 11.

**Figure 12:**
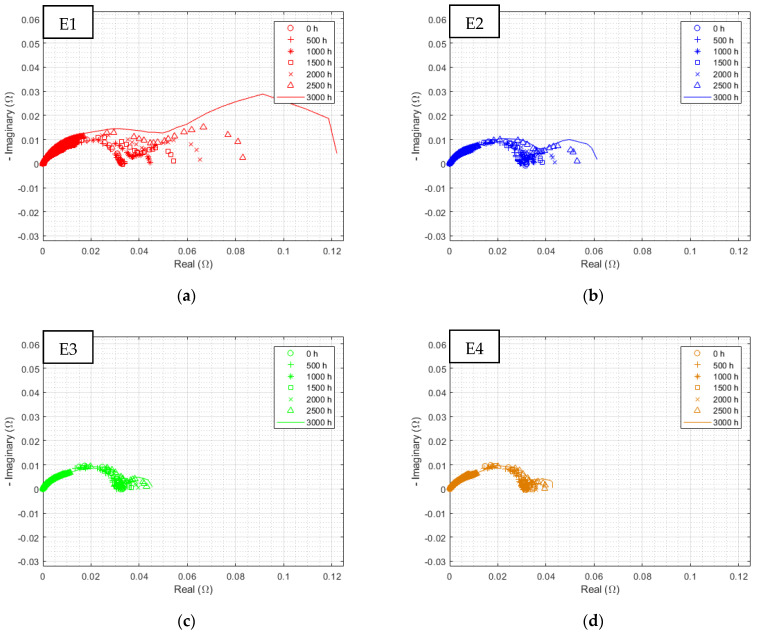
Evolution of EIS at 0.125 A/cm^2^ from 10 kHz to 10 mHz with HFR subtracted over time for electrolyzer: (**a**) E1, (**b**) E2, (**c**) E3, and (**d**) E4, at an ambient pressure of 1 bar with an ambient temperature regulated at 25 °C.

The previous observations of polarization curves were found. There is a small difference between the E3 (sinusoidal 300 Hz current ripple) and E4 (reference) corrected spectra, while an unobserved phenomenon appears at low frequency, mostly for both electrolyzers, withstanding triangular current ripple (E1 and E2). Figure 12 displays all EIS at the highest current density separately for each electrolyzer over the complete endurance test.

No distinctions can be made during the first 500 h. Thereafter, a new semicircle appears on the impedance spectrum of E1, corresponding to an increase in the limitations linked to the low-frequencies phenomena. The diameter of this new EIS signature further increases throughout the test. The same peculiarity can be observed with a reduced impact on E2, and to an even lesser degree on E3 and E4.

**Figure 13:**
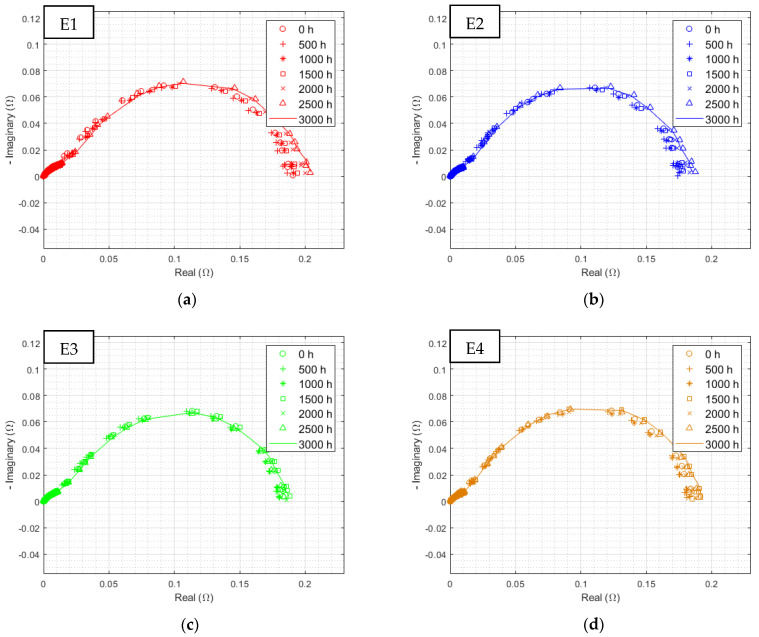
Evolution of EIS at 0.0125 A/cm^2^ from 10 kHz to 10 mHz with HFR subtracted over time for electrolyzer: (**a**) E1, (**b**) E2, (**c**) E3, and (**d**) E4 at an ambient pressure of 1 bar with an ambient temperature regulated at 25 °C.

Figure 13 shows all EIS over time at a lower current density. It can be seen that the diameter of the charge transfer semicircle hardly changes over time. However, even at such a reduced current density, a somewhat attenuated low-frequency phenomenon is again identified.

Such low-frequency events, predominant in the high current densities, are assimilated in the mass transport predicament. These are usually imputed to a two-phase flow regime with the accumulation of gas bubbles and their interaction with water [40,47,48,49]. Similar formations of mass transport semicircles on impedance spectra may be attributed to the lack of water and the dehydration of the membrane [50]. However, dry conditions lead to an HFR increase with the current density, which is not the case in our experiment (as seen in Section 3.2.2). Thus, this behavior should be closely linked to the degradations previously underlined, such as the cationic contamination of the membrane [39] or PTL degradation. Indeed, the modification of PTL porosity [41] or structure [51] results in mass transport limitations.

### 3.3. Reversible Voltage Decrease

**Figure 14:**
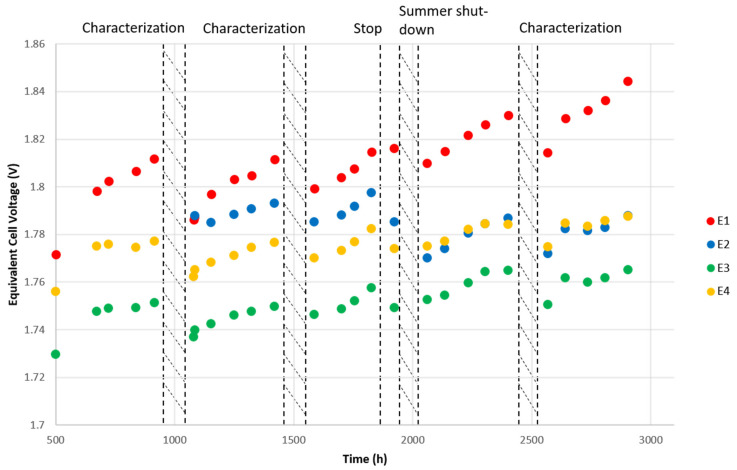
Survey of stack voltages after every refueling. Striped areas correspond to shut-down periods or characterization phases. This measurement was not recorded before 500 h of operation.

As mentioned above, reversible losses are observable on the voltage monitoring (Figure 12). These measurements were taken after every water refilling to ensure the same conditions. It appears that after each characterization phase, or any short stop in operation, cell voltages drop significantly for the same current. This recovery was reported for an interruption as short as 5 min and was accountable for 61% of the global degradation in other studies [42]. With an interruption of one hour before every characterization, we expected to reduce the proportion of reversible losses in our observations. However, Figure 14 shows a better voltage recovery after the characterization phases, equivalent to an 8 h stop before returning to the experiment, than after a short, unexpected interruption of one hour (at approximatively 1850 h of operation). Further investigations of these reversible degradations are in progress.

This phenomenon is closely linked to mass transport limitations [52] and bubble accumulation, essentially within the anode flow-field [43]. These bubbles are supposed to be flushed out during changes in potential, resulting in the observed recovery. Periodically reducing or stopping the input current could be used as a specific dynamic operation to improve the performance and durability of a PEM [13,46].

## 4. Conclusions

Four PEM electrolyzers were operated for 3000 h with different ripple currents characteristic of converters. An increase in the ohmic resistance value over time was observed for all of them. The corrosion and passivation of titanium mesh were most likely responsible for this degradation. Moreover, this phenomenon seemed to be enhanced by triangular current ripples with frequencies of 10 kHz and 1 kHz with an amplitude +/−10% that of the nominal current. Mass transport limitations were clearly observable on the uppermost current density EIS, where a new semicircle appeared after 1000 h of operation and was once again amplified by a triangular current ripple of 10 kHz. These limitations further increased throughout the endurance test. These degradations were most likely linked to the HFR rise and titanium deterioration. The sinusoidal current ripple of 300 Hz did not show any impact on the component performance and lifespan in this study. The activation over-potential phenomenon was not affected by any ripple current. Reversible degradations should be further analyzed to determine to what extent they contribute to the evolution of the total voltage.

The results presented in this paper constitute a primary exploration with the potential to lead to future discussions and experiments. Identical durability tests will soon be conducted on a commercial PEM electrolyzer of 25 cm^2^, with further characterizations to strengthen our results.

## Figures and Tables

**Table 1:** Overvoltage evolution rates at 0.19 A/cm^2^ for 3000 h of operation (μV/h).

Triangular Current Ripple 10 kHz—E1	Triangular Current Ripple 1 kHz—E2	Sinusoidal Current Ripple 300 Hz—E3	Reference Constant Current E4
96.6	34.6	12.6	19.7

**Table 2:** Slopes of linear fittings of the HFR evolution (μΩ/h).

Triangular Current Ripple 10 kHz—E1	Triangular Current Ripple 1 kHz—E2	Sinusoidal Current Ripple 300 Hz—E3	Reference Constant Current E4
10.8	4.6	3.9	5

## Data Availability

The data presented in this study are available on request from the corresponding author.
